# Stress depression and anxiety with xerostomia among young Indian adults

**DOI:** 10.6026/973206300191365

**Published:** 2023-12-31

**Authors:** RM Vatchala Rani, Deepak Kumar, Shiras Singh Dheer, Abhishek Singh Pathania, Madhusudhana Reddy Dasara, Navjeet Kaur

**Affiliations:** 1 Department of Oral Pathology and Microbiology, Faculty of Dentistry, Jamia Millia Islamia, New Delhi, India; 2 Department of Dentistry, Maharaja Suhel Dev Autonomous State Medical College & Maharishi Balark Hospitals, Bahraich, Uttar Pradesh, India; 3 Department of Dentistry, Shrimant Rajmata Vijayaraje Scindia Medical College, Shivpuri, M.P., India; 4 Department of Prosthodontics and Crown & Bridge and Implantology, Dental Officer at ECHS Polyclinic, Jagdalpur, Chattisgarh, India; 5 Department of Oral and Maxillofacial Surgery, Government Dental College and Research Institute, Ballari VIMs Campus Cantonment, Ballari 583014, India; 6 MDS, Department of Conservative Dentistry and Endodontics, Private Practitioner, The Dentist Dental Clinic, Zirakpur, Punjab, India

**Keywords:** Oral health related quality of life (OHRQOL), stress, depression, anxiety, xerostomia, un-stimulated salivary flow rate

## Abstract

The relationship of Oral health related quality of life (OHRQOL), stress, depression, anxiety, with xerostomia, un-stimulated salivary flow rate among young Indian adults is of interest. The first phase involved xerostomia along with salivary flow rate measurements. The Depression, Anxiety and Stress Scale (DASS) the standard questionnaire in this field, was used to assess depression, anxiety, and stress in the second part of the study. Xerostomia has a stronger effect on OHRQOL. Anxiety, stress, and depression are examples of psychological factors that significantly impact xerostomia and the reduction of salivary flow rate.

## Background:

Preserving dental as well as oral wellness is greatly aided by saliva. The fundamental flow rate, or unstimulated flow of saliva rate, ranges from 0.29 to 0.41 millilitres per minute [1,2]. Hypo salivation is defined as a flow rate of less than 0.1 ml/min, which is indicative of hypo-functioning salivary glands [3,4]. Prolonged hypo-salivation-induced xerostomia can lead to a number of oral diseases and conditions, including caries, mucosal ulceration, fissures, tongue depapillation, burning mouth syndrome, periodontal disease and candida infections. These conditions ultimately impact the quality of life associated with oral health (OHRQOL) [5,6]. The word "stress" describes a set of circumstances that cause the brain to react (perceive stress), which in turn triggers the body's physiological fight-or-flight response [7,8]. Anxiety can also be defined as an ambiguous unpleasant and widespread feeling of worry and dread that has an unclear source [9,10]. It is composed of apprehension, powerlessness, and physiological excitement. The manifestations of depression include thoughts, feelings, and behaviours that are indicative of the illness, such as the patient's perpetual emotions of sadness, anxiety and hopelessness [11,12]. Saliva is an extensive mixture of major as well as minor gland secretions from the salivary glands that clears the interior of the mouth, aids in food gnawing, and makes swallowing easier [13,14]. Because saliva acts as a buffer, acids in the mouth are neutralized and teeth are shielded. Saliva possesses antimicrobial qualities and fortifies the mucosal barrier [15,16].In the general population as a whole, there are differences in the prevalence of decreased secretions from salivary gland [17,18]. Generally speaking, xerostomia is more common in women than in men. Salivary gland function decreased over the course of a 15-year study, going from fifteen percent at age of 50 years to six percent at age 65 years [19,20]. Diminished salivation can cause a number of adverse effects, including degeneration of the mucosa, oral candida infections, inflammatory processes of the lining of the mouth (mucositis) and difficulties speaking and chewing [21, 22]. It can aggravate plaque build-up and reduce saliva's ability to act as a buffer. Numerous factors, including anxiety, stress, depressive disorders, age, prior history of treatment with chemotherapy or radiation treatment, pharmaceuticals, and other factors, may have an impact on saliva production [23, 24]. Stress, depression and anxiety are among the various risk factors discussed above that affect saliva. Some scholars have considered these factors because of comparatively strong role of their clinical manifestations and and their treatments in saliva production [25,26]. On the other hand, there is insufficient data to determine how these variables and salivation are related. Some researchers have taken stress into consideration because they believe it may contribute to xerostomia [27]. The majority of xerostomia study is on either elderly patients with pre-existing systemic conditions or patients with clinical or systemic oral diseases. Additionally, there is not enough data to determine whether perceived stress and both objective and subjective xerostomia are related in healthy young adults in India [23-27]. Moreover, the number of studies correlating unstimulated salivary flow rate, xerostomia with OHRQOL in young adults is very low. Therefore, it is of interest to evaluate the relationship of stress, depression, anxiety, xerostomia, unstimulated salivary flow rate, and OHRQOL among young adults.

## Methods and Materials:

## Study design and participants:

## Inclusion and exclusion criteria:

After providing their informed consent and being chosen at random, 494 patients who had been referred to the Dental Hospital were included in the study. The participants were then given a questionnaire that asked about their medical and dental histories in addition to demographic information. Those who were younger than eighteen years, had a past record of systemic illness, having a history of smoking, having a history of any form of medication at the commencement of the research or six months prior, or both were excluded.

## Data collection

This study involved two phases of data collection: the first phase involved xerostomia along with salivary flow rate measurements. There are a few different techniques to gather whole saliva: aspiration, spitting, draining, and using absorbent materials. Spitting and use of absorbent materials are two techniques are normally used. In order to prevent the influence of the human circadian rhythm on salivary flow rates, patients were instructed not consume food or liquids, smoke, or engage in any other oral saliva stimulation of any type, such as brushing, from 90 minutes prior to the collection of saliva specimens. Samples of saliva were taken between eight and nine in the morning. Subsequently, every 60 seconds, the participants were instructed to spit their saliva into designated containers for five minutes. There were two milli-litre syringes available for measuring saliva. Next, in order to evaluate xerostomia (the sensation of having a dry mouth), the patients' symptoms were noted using a specially designed form. Then, based on the information gathered, the study participants were divided into four separate categories:

Category 1: Unstimulated salivary flow rate <0.1 mL/min with xerostomia: 120 subjects

Category 2: Unstimulated salivary flow rate <0.1 mL/min without xerostomia: 120 subjects

Category 3: Unstimulated salivary flow rate >0.1 mL/min with xerostomia: 118 subjects

Category 4: Unstimulated salivary flow rate >0.1 mL/min without xerostomia: 136 subjects

Participants in the salivary flow rate evaluation were told the day before to abstain from eating, drinking, brushing their teeth, and smoking for sixty minutes on the day of information gathering. To minimize fluctuations in saliva secretion related to the circadian cycle and to standardize the protocol, all specimens of saliva were gathered between 8am and 10 am. All participants were instructed to sit up straight in an empty space in order to provide a saliva sample. Saliva that had not been stimulated was gathered using the draining technique and placed in a pre-weighed plastic package. The participant was directed to perform a preliminary swallow to clear their oral cavity of any leftover saliva. Participants were told to refrain from swallow the saliva again until the researcher notified them to do so.

The first swallow was selected as the starting point for a stopwatch, and the participant was told that they would be notified by a bell to indicate when it was time to cease their salivating into the container after five minutes. As soon as saliva began to accumulate, the participant began to drool into the container, holding their head slightly tilted and their mouth partially open to facilitate the drooling. The container was weighed one more time after the collection period. The weight of the saliva was calculated in grammes by deducting the total weight of the container both before and after the saliva was collected. The unstimulated salivary flow was computed by dividing the total amount of saliva by the time for entire collection considering 1 g of saliva is equivalent to 1 ml.

The Depression, Anxiety and Stress Scale (DASS)[21,25], the standard questionnaire in this field, was used to assess depression, anxiety, and stress in the second part of the study. Initially, the questionnaire had been modified into local language by two professionals in oral medicine, and the accuracy of its information was verified. The reliability was evaluated using Cronbach's alpha coefficient, which was 0.937, 0.808, and 0.899 for the areas of anxiety, stress, and depression, respectively. For the three areas, the average Cronbach's alpha coefficient was 0.798.

There are 42 questions on the questionnaire, divided into three sections: anxiety, stress, and depression. There are fourteen questions in each section, codified from zero to three (never = zero, a little = one, occasionally = 2, and always = 3). Each part has an acceptable score range of 0-42. Following completion of the questionnaire, each person's anxiety, stress, and depression scores were classified as normal state, mild state, moderate state, severe state, or very severe state based on reference ranges of values (Table 1).

For OHRQOL, a condensed form of the Oral Health Impact Profile (S-OHIP), a trustworthy and validated questionnaire with 14 questions, was utilised. The total score falls between 0 and 56. A higher score indicates a worse OHRQOL and a larger oral impact. The different levels of OHRQOL was poor, fair, good, very good, excellent (Table 2).

## Data analysis:

Both descriptive as well as analytical evaluations were implemented in this investigation. Quantitative data was analyzed using descriptive statistics like standard deviations and means, while qualitative variables were analyzed using frequencies as well as percentages. Additionally, Fisher's exact statistical test and chi-squared statistical test were used for the purpose of analysis. P<0.05 was used to indicate statistical significance. With SPSS 18, statistical calculations were performed.

## Results:

The frequency of study participants with no depression was 10.4% in category 1, 21.9% in category 2, 25.9% in category 3, 33.5% in category 4. The frequency of study participants with mild depression was 15.4% in category 1, 25.6% in category 2, 30.2% in category 3, 36.3% in category 4. The frequency of study participants with moderate depression was 16.4% in category 1, 18.4% in category 2, 14.8% in category 3, 10.3% in category 4. The frequency of study participants with severe depression was 25.3% in category 1, 21.3% in category 2, 18.5% in category 3, 14.4% in category 4. The frequency of study participants with very severe depression was 32.5% in category 1, 12.8% in category 2, 19.6% in category 3, 5.5% in category 4.It was observed that frequency of severe and very severe depression was high in study participants with reduced unstimulated saliva flow rate with xerostomia, followed by normal unstimulated saliva flow rate with xerostomia. The correlation of unstimulated saliva flow rate and xerostomia with depression was significant statistically (Table 3).

The frequency of study participants with no stress was 15.4% in category 1, 25.8% in category 2, 27.8% in category 3, 32.7% in category 4. The frequency of study participants with mild stress was 20.4% in category 1, 21.7% in category 2, 29.3% in category 3, 35.1% in category 4. The frequency of study participants with moderate stress was 21.4% in category 1, 20.5% in category 2, 18.9% in category 3, 09.5% in category 4. The frequency of study participants with severe stress was 19.3% in category 1, 18.4% in category 2, 14.7% in category 3, 10.2% in category 4. The frequency of study participants with very severe stress was 23.5% in category 1, 13.8% in category 2, 16.4% in category 3, 6.5% in category 4.It was observed that frequency of severe and very severe stress was high in study participants with reduced unstimulated saliva flow rate with xerostomia, followed by normal unstimulated saliva flow rate with xerostomia. The correlation of unstimulated saliva flow rate and xerostomia with stress was significant statistically (Table 4).

The frequency of study participants with no anxiety was 1.5% in category 1, 11.6% in category 2, 1.2% in category 3, 51.4% in category 4. The frequency of study participants with mild anxiety was 2.4% in category 1, 12.6% in category 2, 1.6% in category 3, 29.3% in category 4. The frequency of study participants with moderate anxiety was 15.4% in category 1, 25.6% in category 2, 5.3% in category 3, 12.4% in category 4. The frequency of study participants with severe anxiety was 29.3% in category 1, 19.5% in category 2, 30.2% in category 3, 2.4% in category 4. The frequency of study participants with very severe anxiety was 51.4% in category 1, 30.6% in category 2, 61.7% in category 3, 4.5% in category 4.It was observed that frequency of severe and very severe anxiety was high in study participants with reduced unstimulated saliva flow rate with xerostomia, followed by normal unstimulated saliva flow rate with xerostomia. The correlation of unstimulated saliva flow rate and xerostomia with anxiety was significant statistically (Table 5).

The frequency of study participants with poor OHRQOL was 52.4% in category 1, 31.6% in category 2, 62.7% in category 3, 5.5% in category 4. The frequency of study participants with fair OHRQOL was 28.3% in category 1, 18.5% in category 2, 31.2% in category 3, 1.4% in category 4. The frequency of study participants with good OHRQOL was 16.4% in category 1, 25.6% in category 2, 7.3% in category 3, 13.4% in category 4. The frequency of study participants with very good OHRQOL was 3.3% in category 1, 11.5% in category 2, 2.7% in category 3, 28.2% in category 4. The frequency of study participants with excellent OHRQOL was1.6% in category 1, 11.7% in category 2, 1.3% in category 3, 51.5% in category 4.It was observed that frequency of poor and fair OHRQOL was high in study participants with reduced unstimulated saliva flow rate with xerostomia, followed by normal unstimulated saliva flow rate with xerostomia. The correlation of unstimulated saliva flow rate and xerostomia with OHRQOL was significant statistically (Table 6).

## Discussion:

It is of interest to evaluate the relationship of stress, depression, anxiety, xerostomia, unstimulated salivary flow rate, and OHRQOL among young adults. The frequency of study participants with severe stress was 19.3% in category 1, 18.4% in category 2, 14.7% in category 3, 10.2% in category 4. The frequency of study participants with very severe stress was 23.5% in category 1, 13.8% in category 2, 16.4% in category 3, 6.5% in category 4.It was observed that frequency of severe and very severe stress was high in study participants with reduced unstimulated saliva flow rate with xerostomia, followed by normal unstimulated saliva flow rate with xerostomia. The correlation of unstimulated saliva flow rate and xerostomia with stress was significant statistically. It is known that depression and psychological stress are important factors in both raising the probability of xerostomia and decreasing salivary flow rate [5,8]. In this regard, xerostomia and unstimulated flow rates of saliva less than 0.1 mL/min are more common for people with anxiety, stress, and depressive disorders, according to previous study evaluation of 1202 study participants in three distinct categories [7-10]. Our results are in line with those of previous study who demonstrated a significant relationship between insomnia and mood disorders as well as decreased salivary flow rates. This study also showed that xerostomia and burning sensation are more common in women than in men who are depressed. It is also shown that stress can cause low functioning of the salivary glands, which lowers the amount of saliva produced. Researchers discovered a significant correlation among exam stress and a drop in the rate of saliva flow and overall salivary concentrations of proteins [9-12]. In our study, the frequency of study participants with severe depression was 25.3% in category 1, 21.3% in category 2, 18.5% in category 3, 14.4% in category 4. The frequency of study participants with very severe depression was 32.5% in category 1, 12.8% in category 2, 19.6% in category 3, 5.5% in category 4.It was observed that frequency of severe and very severe depression was high in study participants with reduced unstimulated saliva flow rate with xerostomia, followed by normal unstimulated saliva flow rate with xerostomia. The correlation of unstimulated saliva flow rate and xerostomia with depression was significant statistically. Data also indicated that psychological disorders, including depression, confusion, memory loss, and insomnia, affected 41.9% of the subjects who reported feeling dry mouth [13-17]. However, other potential reactions were hypothesized by certain researchers using salivary proteins that rise in response to acute stress stimuli; however, salivary flow rate was unaffected by acute stress (public discourse) [18-20]. A number of factors, including the possibility that gender and age have an impact on salivary gland function could be responsible for this discrepancy in the results. Interestingly a previous research did not include any female subjects to prevent the influence of female hormone production on cortisol levels. Additionally, the duration and intensity of stress have varying effects on immune function [21,22]. The frequency of study participants with severe anxiety was 29.3% in category 1, 19.5% in category 2, 30.2% in category 3, 2.4% in category 4. The frequency of study participants with very severe anxiety was 51.4% in category 1, 30.6% in category 2, 61.7% in category 3, 4.5% in category 4.It was observed that frequency of severe and very severe anxiety was high in study participants with reduced unstimulated saliva flow rate with xerostomia, followed by normal unstimulated saliva flow rate with xerostomia. The correlation of unstimulated saliva flow rate and xerostomia with anxiety was significant statistically. Acute stress, such as exams, can lower salivary levels, but mild stress, such as PMS (premenstrual syndrome), has little impact on salivary flow rate, as demonstrated by previous research [23- 26]. The current study evaluated "stress" as a general psychological disorder; however, due to the lack of specific data on the characteristics of stress, anxiety, and depression, we were unable to evaluate the various types of stress in terms of their severity or acuity [21-24]. This explains why the findings of the two subsequent investigations differ from those of this study. The impact of stressful circumstances in two distinct categories in a different study (with as well as without anxiety) is known. They discovered a strong correlation between the rise in cortisol levels and hypo salivation [20-24]. In their research, authors found a strong correlation between psychological variables (like anxiety) and a dry mouth or taste disorders. Similar findings to the current study were reported by both of these researchers [21- 25]. The frequency of study participants with poor OHRQOL was 52.4% in category 1, 31.6% in category 2, 62.7% in category 3, 5.5% in category 4. The frequency of study participants with fair OHRQOL was 28.3% in category 1, 18.5% in category 2, 31.2% in category 3, 1.4% in category 4. It was observed that frequency of poor and fair OHRQOL was high in study participants with reduced unstimulated saliva flow rate with xerostomia followed by normal unstimulated saliva flow rate with xerostomia. The correlation of unstimulated saliva flow rate and xerostomia with OHRQOL was significant statistically. It is known that OHRQOL was significantly decreased by xerostomia [25,26]. According to previous research there is a significant correlation between OHRQOL and xerostomia presence or absence, with poor OHRQOL occurring more frequently as compared to that of our study [27]. This discrepancy might result from our study's participation from a younger and healthier population. The limitations is that a control group that consisted of people who were not under stress and who were matched for age and gender was not used, which would have allowed us to better understand the relationship between stress as well as xerostomia.

## Conclusion:

Data shows that xerostomia has a stronger effect on OHRQOL. Anxiety, stress, and depression are examples of psychological factors that significantly impact xerostomia and the reduction of salivary flow rate.

## Figures and Tables

**Table 1:** Range of scores for different states of depression, anxiety and stress

	**Normal**	**Mild**	**Moderate**	**Severe**	**Very severe**
Depression	0-9	Oct-13	14-20	21-27	28+
Stress	0-14	15-18	19-25	26-33	34+
Anxiety	0-7	08-Sep	Oct-14	15-19	20+

**Table 2:** Range of scores for quality of life

**Poor**	**Fair**	**Good**	**Very Good**	**Excellent**
0-10	Nov-21	22-33	34-45	46-56

**Table 3:** Frequency of different stages of depression

	**Normal**	**Mild**	**Moderate**	**Severe**	**Very severe**
Category 1	10.40%	15.40%	16.40%	25.30%	32.50%
Category 2	21.90%	25.60%	18.40%	21.30%	12.80%
Category 3	25.90%	30.20%	14.80%	18.50%	19.60%
Category 4	33.50%	36.30%	10.30%	14.40%	5.50%
P value			0.001		

**Table 4:** Frequency of different stages of stress

	**Normal**	**Mild**	**Moderate**	**Severe**	**Very severe**
Category 1	15.40%	20.40%	21.40%	19.30%	23.50%
Category 2	25.80%	21.70%	20.50%	18.40%	13.80%
Category 3	27.80%	29.30%	18.90%	14.70%	16.40%
Category 4	32.70%	35.10%	9.50%	10.20%	6.50%
P value			0.001		

**Table 5:** Frequency of different stages of anxiety

	**Normal**	**Mild**	**Moderate**	**Severe**	**Very severe**
Category 1	1.50%	2.40%	15.40%	29.30%	51.40%
Category 2	11.60%	12.60%	25.60%	19.50%	30.60%
Category 3	1.20%	1.60%	5.30%	30.20%	61.70%
Category 4	51.40%	29.30%	12.40%	2.40%	4.50%
P value			0.001		

**Table 6:** Frequency of different stages of OHRQOL

	**Poor**	**Fair**	**Good**	**Very Good**	**Excellent**
Category 1	52.40%	28.30%	16.40%	3.30%	1.60%
Category 2	31.60%	18.50%	26.60%	11.50%	11.70%
Category 3	62.70%	31.20%	7.30%	2.70%	1.30%
Category 4	5.50%	1.40%	13.40%	28.20%	51.50%
P value			0.001		
